# Immunotherapy and Targeted Therapies Efficacy in Thymic Epithelial Tumors: A Systematic Review

**DOI:** 10.3390/biomedicines11102722

**Published:** 2023-10-08

**Authors:** Apostolos C. Agrafiotis, Mariana Brandão, Thierry Berghmans, Valérie Durieux, Christiane Jungels

**Affiliations:** 1 Department of Thoracic and Vascular Surgery, Antwerp University Hospital, University of Antwerp, B-2650 Edegem, Belgium; 2 European Lung Cancer Working Party (ELCWP), 1070 Brussels, Belgium; 3 Thoracic Oncology Unit, Institut Jules Bordet, Hôpital Universitaire de Bruxelles, Université Libre de Bruxelles, 1070 Brussels, Belgium; 4 Bibliothèque des Sciences de la Santé, Université libre de Bruxelles, 1070 Brussels, Belgium; 5 Department of Oncological Medicine, Institut Jules Bordet, Hôpital Universitaire de Bruxelles, Université Libre de Bruxelles, 1070 Brussels, Belgium

**Keywords:** immunotherapy, targeted therapies, thymoma, thymic carcinoma, tumor microenvironment

## Abstract

Background: Thymic epithelial tumors (TET) are rare neoplasms of the anterior mediastinum. Surgery is the mainstay treatment for resectable TET, whereas systemic treatments are reserved for unresectable and metastatic tumors. The development of new treatments, such as immune checkpoint inhibitors (ICI) and targeted therapies, with promising results in other types of solid tumors, has led to the investigation of their potential efficacy in TET. The study of tumor microenvironments (TME) is another field of investigation that has gained the interest of researchers. Taking into account the complex structure of the thymus and its function in the development of immunity, researchers have focused on TME elements that could predict ICI efficacy. Materials and Methods: The primary objective of this systematic review was to investigate the efficacy of ICI in TET. Secondary objectives included the toxicity of ICI, the efficacy of targeted therapies in TET, and the evaluation of the elements of TME that may be predictive factors of ICI efficacy. A literature search was conducted in February 2023 using the Ovid Medline and SciVerse Scopus databases. Results: 2944 abstracts were retrieved, of which 31 were retained for the systematic review. Five phase II and one retrospective study assessed ICI efficacy. The overall response rate (ORR) varied from 0% to 34%. Median progression-free survival (PFS) ranged from 3.8 to 8.6 months, being lower in thymic carcinoma (TC) (3.8–4.2 months). Median overall survival (OS) ranged from 14.1 to 35.4 months. Treatment-related adverse events occurred in 6.6% to 27.3% of patients. Sixteen studies assessed targeted therapies. The most active molecule was lenvatinib, with 38% ORR in patients with TC while no activity was detected for imatinib, erlotinib plus bevacizumab, and saracatinib. Ten studies assessed TME elements that could predict ICI efficacy. Four studies focused on the tumor-infiltrating immune cells suggesting improved outcomes in patients with TC and high tumor-infiltrating lymphocyte densities. Another study showed that CD8+, CD20+, and CD204+ tumor-infiltrating immune cells in cancer stroma might be prognostic biomarkers in TC. Another study identified the immune-related long non-coding RNAs as a predictor of response to ICI. Tumor mutational burden was identified as a predictive factor of ICI efficacy in one study. Conclusions: Despite study heterogeneity, this review shows that ICI could be a therapeutic option for selected patients with TET that are not amenable to curative radical treatment after first-line chemotherapy.

## 1. Introduction

Thymic epithelial tumors (TET), which account for 15% of all anterior mediastinal tumors, are uncommon neoplasms of the prevascular mediastinum [1]. They are derived from the epithelial cells of the thymus and are categorized in relation to the fraction of the non-tumoral lymphocytic part, and to their resemblance to normal thymic architecture. This heterogeneous group of neoplastic lesions includes thymomas and thymic carcinomas (TC). The 2015 revised World Health Organization (WHO) classification system classified TET as A, AB, B1, B2, and B3 thymoma, and TC [2]. Thymomas may present an indolent course and, for that reason, were formerly considered benign neoplasms. However, they are nowadays classified as malignant lesions. The overall prognosis is good for thymomas that are amenable to complete surgical resection [3]. On the contrary, TC are characterized by a more clinically aggressive behavior [3,4]. The majority of patients are eligible for surgical treatment, which may be combined with adjuvant radiation. Chemotherapy is prescribed to patients with unresectable TET, at advanced stages (stages III–IV according to either the Masaoka–Koga or the ITMIG classification), or for recurring diseases [4,5]. Platinum-based chemotherapy is the most often used regimen but there is no formal consensus on the best regimen due to the absence of randomized trials in these rare entities [3,4,5].

Recently, the introduction of new treatments, such as immune checkpoint inhibitors (ICI), with promising results in other types of solid tumors, has led researchers to investigate their potential efficacy in TET [6,7,8,9,10,11,12,13]. However, there is limited evidence about their clinical advantage.

Similarly, there is an increased interest in the detection of molecular alterations that could be modified by targeted therapies [14,15,16,17,18,19,20,21,22,23,24,25,26,27,28,29]. New drugs are selectively targeting the pathways that play an important role in oncogenesis, tumor growth, and proliferation. The rarity and the histological heterogeneity of TET constitute a major obstacle in the conduct of large-scale trials and, until now, the majority of evidence has derived from case reports and small case series.

Another research field that has gained recent interest is that regarding the tumor microenvironment (TME) [30]. The TME consists mainly of endothelial cells of the vascular epithelium, cells of innate and adaptive immunity, fibroblasts, pericytes, signal-carrying molecules, and the extracellular matrix. A close dynamic relationship exists between the tumor and its microenvironment [31,32,33]. This complex interaction is a key element in oncogenesis, growth, and tumor spread. Taking into account the complex structure of the thymus and its function in the development of immunity, researchers are focusing on the TME elements that could predict ICI efficacy [32,33]. The expression of PD-L1 on tumor cells contributes to the prediction of clinical efficacy of ICI in some tumor types, such as non-small cell lung cancer, which take advantage of the crucial roles played by the PD-L1/PD-1 and CTLA4/CD80/CD86 axes in the evasion of immune surveillance. As a result, the distinct composition of TME within various TET histological categories, along with the variations seen among them in the PD-L1 expression patterns, constitutes an essential component of their biological background and directly regulate both the response to checkpoint-inhibitory receptor blockade and the predisposition to autoimmune disease [31,32,33].

The objective of this systematic review was to assess the most recent data on the effectiveness of immunotherapy treatments and the advantages of targeted therapies against potentially treatable mutations in TET. Additionally, components of the TME that might serve as indicators of ICI effectiveness were evaluated.

## 2. Material and Methods

The literature search, which was designed by a scientific librarian (VD) with competence in medical literature research, was carried out in February 2023 utilizing the Ovid Medline and SciVerse Scopus databases. The search parameters were converted into MeSH terms and free-text keywords, which were then used to search for specific information in titles, abstracts, keywords, and substance names (where applicable) in Medline and titles, abstracts, and keywords in Scopus (Appendix A). The resulting citations were exported from Medline and Scopus into a reference manager software (EndNote version X9) to remove any duplicates, and then in a dedicated systematic literature reviews system (https://rayyan.ai, accessed on 2 February 2023) for the selection process. The researchers worked simultaneously by composing two pairs (ACA and CJ, TB and MB) that ran the initial article selection independently. In the first step, articles were selected if deemed eligible based on the title and abstract content. The final selection was performed after reading the full-text article. The selections of both groups of reviewers were compared and the mutually selected papers represented the total eligible papers to be analyzed. Any discrepancies between the two groups of researchers were resolved after a consensual discussion. The selected articles’ references were examined to detect any missing potentially eligible publications.

The inclusion criteria were the following:

Only articles in English, French, or Dutch were considered. There was no selection based on the year of publication.

(1) Phase II/III clinical trials and retrospective series (>14 patients according to Simon’s design) [34,35] assessing ICI in TET and reporting at least one of the following clinical outcomes:
  - progression-free survival (PFS), defined as the time from randomization to disease progression or death from any cause;
  - overall survival (OS), defined as the time from randomization until death from any cause;
  - objective response rate (ORR), defined as the proportion of patients who achieved an objective response (partial or complete according to the Response Evaluation Criteria in Solid Tumors (RECIST));
  - all grade or grade ≥ 3 treatment-related adverse events.
(2) Phase I/II/III clinical trials and retrospective series (>14 patients according to Simon’s design) assessing targeted therapies against an oncogenic driver mutation or translocation (*EGFR, cKIT, KRAS, ALK, BRAF, PDGFR, HER2, MET etc.*).
(3) Experimental cohort studies investigating any of the following:
  − TME of TET, % of PD-L1 expression in TET or tumor mutational burden (TMB) AND prediction of ICI efficacy.

Phase I trials concerning different types of tumors, even including TET, were not considered.

The following data were extracted: study characteristics (design, patient selection), patients’ characteristics (gender, age, previous treatments), stage and histology, treatments and clinical outcomes (number of arms, hazard ratio (HR), and 95% confidence interval (CI) for PFS and OS, overall response rate (ORR), number of patients with grade 3 or greater adverse events (AEs).

The main judgment criterion was ORR. Secondary judgment criteria were PFS, OS (median and at specific time-point: 1–2–5 years), and grade 3–5 AEs.

### Data Synthesis

Given the high heterogeneity in the selected studies in terms of inclusion criteria, treatments, and data presentation, a quantitative analysis was not performed.

## 3. Results

**Figure 1:**
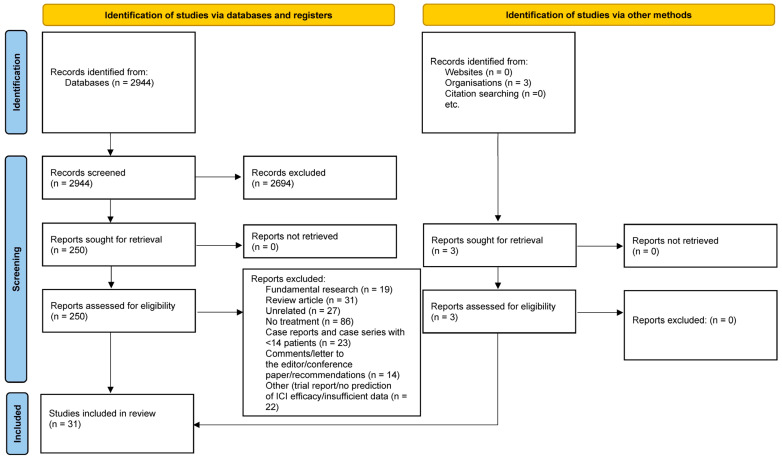
PRISMA flowchart demonstrating the search strategy.

A total of 2944 abstracts were retrieved through the search equation, of which 31 were eligible for the systematic review. The PRISMA flowchart is depicted in Figure 1.

Six trials assessed ICI efficacy in TET [9,13,36,37,38,39,40,41] (Table 1 and Table 2). Five were phase II trials whereas the last was a retrospective cohort with 77 patients enrolled. All were recently published, from 2018 to 2023. Four studies were multicentric and two were elaborated in a single center. The administered drug was pembrolizumab in two studies and nivolumab in another two studies. In one study the ICI avelumab was combined with the anti-angiogenic agent axitinib. In the last study, different PD-1 inhibitors (anti-PD-1: nivolumab, pembrolizumab, sintilimab, camrelizumab, tislelizumab, and toripalimab) were used. The median number of patients was 37 (range 15–77). ORR was the primary endpoint of four phase II trials and the PFS rate at six months of the fifth one. In half of the studies, only patients with TC were included whereas patients with thymomas and TC were assessed in the remaining studies. The Masaoka–Koga classification was used throughout the trials. All patients presented with stage III (which were not candidates for curative surgical resection) or IV (IVa and/or IVb). The median follow-up duration was 14.9 months (range 13.3–22.4 months). The ORR varied from 0% to 34%. In trials exclusively enrolling patients with TC, the ORR was 0% to 22.5%. The mPFS ranged from 3.8 to 8.6 months overall, being 3.8 to 4.2 months in TC. The mOS ranged from 14.1 to 35.4 months. Treatment-related AE occurred in 6.6% to 27.3% of patients (Table 2).

Sixteen studies testing targeted therapies were deemed eligible for further analysis [23,25,42,43,44,45,46,47,48,49,50,51,52,53,54]. There were thirteen phase II trials, two retrospective studies, and one prospective cohort (Table 3 and Table 4). There were four single-center studies, five studies were conducted in two centers and the remaining seven studies were multicenter trials. Different molecules with various actions were evaluated. The following molecules were assessed:

Regorafenib, a VEGFR-PDGFR-FGFR inhibitor;

Apatinib and anlotinib that have a function of VEGFRs, KIT, PDGFRs TKI;

Sunitinib, a VEGFRs, KIT, PDGFRs TKI;

Buparlisib, a pan-PI3K inhibitor;

Saracatinib, a Src inhibitor;

Cixutumumab, an IGF-1R inhibitor;

Everolimus, an mTOR inhibitor;

Belinostat, a pan-HDAC inhibitor;

Gefitinib and erlotinib with a function of EGFR inhibitors;

Imatinib which is a BCR-ABL TKI;

Milciclib, a pan-cyclin d-dependent kinase inhibitor;

Lenvatinib, a multi-targeted inhibitor of VEGFR, FGFR, RET, c-Kit, and other kinases. Publication years ranged from 2008 to 2023, and the number of enrolled patients varied from 14 to 72. The median follow-up duration ranged from 15.5 to 46 months. Most of the studies (13 out of 16) enrolled patients with both thymomas and TC. In all studies, patients were treated with at least one prior chemotherapy scheme treatment. In seven studies, the ORR was the primary endpoint and ranged from 0% to 38%. The highest ORR (38%) was observed in patients with TC treated with lenvatinib, and mOS was not reached. In three studies with imatinib, a combination of erlotinib and bevacizumab, and saracatinib, the ORR was 0%. Grade 3–4 treatment-related AEs varied according to the molecule and are reported in Table 4.

Ten studies assessed TME elements that could predict the efficacy of ICI [55,56,57,58,59,60,61,62,63,64]. One study was published in 2011 and the others from 2019 to 2022. Three studies were based on resected TC specimens (10, 32, and 39 patients). Four studies included mixed histologies (thymomas and TC) with 21, 22, 31, and 33 specimens. In three studies, TETs were included among different cancer types. The results of these different studies are detailed in Table 5. Four studies focused on the tumor-infiltrating immune cells suggesting improved outcomes in patients with TC and high tumor-infiltrating lymphocyte densities [55,57,59,60]. Another one showed that CD8+, CD20+, and CD204+ tumor-infiltrating immune cells in cancer stroma might be prognostic biomarkers in TC [58]. Another study identified the immune-related long non-coding RNAs as a predictor of response to immunotherapy [56]. A Chinese study identified TMB as a predictive factor of ICI efficacy [63]. Researchers from South Korea assessed the first-week proliferative response of PD-1+ CD8+ T cells as a predictive marker [61]. The immunological pathways predisposing to irAE are the subject of another study [62].

## 4. Discussion

TETs are rare and histologically heterogeneous tumors. However, they represent the majority of neoplasms located in the anterior (prevascular) mediastinum. Surgical resection is the treatment of choice for early-stage and resectable tumors, whereas there is no consensus about the best systematic treatments for advanced diseases. This systematic review presents updated data on the clinical activity of ICI and targeted therapies in advanced and metastatic thymoma and TC. It provides a comprehensive review of the existing evidence, which has, until now, been represented mainly by anecdotal case reports and small case series.

### 4.1. Immune Checkpoint Inhibitors in TET

Immunotherapy has drawn the attention of researchers who are looking into its effectiveness in TET because it has demonstrated encouraging effects in other solid tumors. The crucial function of the thymus gland in the formation of adaptive immune responses makes the TET example fascinating [65]. ICI enhances the immune response against tumors but may also trigger immune-related adverse events (irAE). Accordingly, the activity and toxicity of ICI in clinical trials are emphasized in this systematic review. Preliminary encouraging clinical results are reported with ORR up to 34%, mPFS ranging from 3.8 to 8.6 months, and mOS between 14.1 and 35.4 months. A list of ongoing trials derived from clinicaltrials.gov is shown in Table 6.

However, substantial toxicity is observed in 6.6% to 27.3% of patients, which is a significant barrier to their routine and widespread use. IrAEs generally carry a tolerable level of morbidity but occasionally result in fatal outcomes (varying from 0.36% to 1.23%). Lethal toxicities are less frequent with anti-PD-1 and anti-PD-L1 antibodies, in comparison with anti-CTLA-4 antibodies and certainly for combined administrations (anti-PD-1/PD-L1 plus anti-CTLA-4) [36,66,67]. Currently, ICI (pembrolizumab, nivolumab, and avelumab) must only be used in clinical studies, as the most appropriate group of patients, those with reduced risk of irAEs and with the best therapeutic benefit, must yet be defined.

Immunotherapy agents, unlike those used in conventional cytotoxic therapy, work therapeutically by inducing the anti-tumor immune response, which is based on the immunoregulative process that takes place between cancer cells and the TME. In numerous cancer types, attempts to link PD-L1 expression in tumor cells and ICI effectiveness have shown inconsistent results [58]. PD-L1 expression in thymic epithelial malignancies has been documented in earlier research, but its application as a diagnostic biomarker in TET is not well understood. Therefore, more precise biomarkers and more pertinent predictive features for the identification of individuals who will potentially benefit from ICIs are needed to guide patients’ selection [55].

The thymus is an organ in charge of the evolution of adaptive immunity. Thymus cell lymphocytes or T cells, which are crucial components of adaptive immune function, mature in the thymus. More specifically, the complex thymic structure provides a special microscopic environment that directs thymocyte maturation and instructs T cells to develop self-tolerance [65]. More lymphocytes may boost the likelihood of a successful application of ICI because they fight cancer by increasing cytotoxic lymphocytes [59]. ICI effectiveness in TC patients is supported by improved outcomes in patients with high tumor-infiltrating lymphocyte densities [60]. Effector cells targeting cancer cells, including CD8+ cells, are a predictive marker for ICI activity [68]. The balance between effector and suppressor cells may be crucial for the TME function and might serve as a prognostic and predictive biomarker for ICI [69]. Previous studies have suggested that effector cells, such as CD8+ lymphocytes, are favorable prognostic indicators among patients with TC, but these data are inconsistent [57,70]. Sato et al. have demonstrated that CD8+, CD20+, and CD204+ tumor-infiltrating immune cells in cancer stroma might be prognostic biomarkers in TC. More specifically, high mean numbers of stromal CD8+, CD20+, and FOXP3+ cells have been shown to be significantly associated with favorable prognosis, whereas high CD204+ cell density tended to be correlated with poor prognosis [58]. Shim et al. have confirmed these findings, with a link between better TC prognosis and higher density of stromal CD20+ cells (B lymphocytes) [57]. These results suggest that thymic malignancy differs from other cancer types in the influence of CD20+ cells and that the density of CD20+ tumor-infiltrating immune cells in stromal lesions has to be examined. This opens the door to the investigation of immunotherapy approaches targeting B cell immunity in TC [57].

Other arguments suggest the important role of TME in predicting ICI activity or toxicity in TET. Su et al. have developed an immune-related long noncoding RNAs classifier to pinpoint the response in patients with TET. As the authors advocate, long noncoding RNAs can control the immune response by controlling homeostasis, TME, anti-inflammatory agents, and immune cell activity [56]. Six prognosis-related immune-related long noncoding RNAs (AC004466.3, AC138207.2, AC148477.2, AL450270.1, HOXB-AS1, and SNHG8) were selected to build an immune-related long noncoding RNAs classifier. According to these authors, their model can be used to forecast outcomes, the degree of immune infiltration, and the effectiveness of immunotherapy in patients diagnosed with TETs. It may also help with individualized immunotherapy counseling.

Kim et al. assessed the first-week proliferative response of PD-1+ CD8+ T cells as a predictive marker of tumor responses to anti-PD-1 therapy and clinical outcomes in patients with TETs. The proliferative response after anti-PD-1 therapy was evaluated by the fold-change in the percentage of Ki-67+ cells among PD-1+ CD8+ T cells on day 7 (Ki-67 D7/D0). In the cohort of patients with TETs, Ki-67 D7/D0 was found to be significantly higher in patients with durable clinical benefits than in those with no durable benefits [61]. However, Ki-67 D7/D0 significantly predicted OS in patients with non-small cell lung cancer, but not in patients with TETs.

Chen et al. investigated the association between protein kinase, DNA-activated, catalytic subunit (*PRKDC)* mutations and TMB, TME, and response to ICI on solid tumor samples collected from 3877 patients that underwent a panel-based next-generation sequencing assay [63]. *PRKDC* is an important gene for DNA double-strand break repair and central T-cell tolerance. *PRKDC* mutation is one of the significant factors linked to increased TMB, inflamed TME, and greater responsiveness to ICI. It frequently appears to co-exist with defects in other DNA damage repair pathways. However, their specificity in TET needs to be validated in larger-scale trials.

Yip1 interacting factor homolog B (YIF1B) is a membrane protein that belongs to the FinGER protein family. It is involved in the endoplasmic reticulum (ER)-to-Golgi trafficking [71]. Recent research has demonstrated its role in serotonin-induced cancerogenesis. Liu et al. found a positive relationship between YIF1B expression and immune cell infiltration in several cancer types, and YIF1B expression was also found to be positively correlated with TMB, microsatellite instability, and methylation in some cancer types, linking its expression to a possible evaluation of therapy response [64].

In another study, peripheral blood T-cell characteristics are linked to the emergence of irAEs following anti-PD-1 medication and four different patient subgroups are defined: Th17-related, TNF-related, CD8-related Treg-compensated, and CD8-related Treg-uncompensated. Patients with severe irAEs presented a significantly lower fold increase in the frequency of effector regulatory T (eTreg) cells after anti-PD-1 treatment, a higher proportion of T helper-17 (Th17) and T helper-1 cells in the beginning, and an increased fraction of Ki-67+ cells among PD-1+ CD8+ T cells post treatment. Various irAE subtypes may have unique underlying immunological processes [62]. Early assessment of immune responses may also have clinical implications for irAE prediction.

### 4.2. Targeted Therapies in TET

Compared with thymomas, TC exhibits more somatic mutations in cancer-related genes [72]. Thus, it is reasonably expected that thymoma and TC may have distinct responses to targeted therapies. Different potential targets have been identified in thymoma and/or TC that are reported hereafter.

Except for a few isolated case reports in Asian individuals, somatic activating *EGFR* mutations are relatively uncommon in thymic malignancies [73,74]. EGFR protein overexpression is present in approximately 70% of thymomas and 50% of TC without any relationship with the histologic subtype [75,76]. About 20% of thymic malignancies exhibit *EGFR* gene amplification by fluorescence in situ hybridization (FISH), most frequently in type B3 thymoma and TC, related to more advanced stage and capsule invasion.

KIT immunohistochemical positivity can be seen in up to 73–86% of TC but only in 2% of thymomas [76,77]. As KIT is a target in other tumor forms, most notably in gastrointestinal stromal tumors, this variation in tumor biology results in a definite difference in therapeutic approaches between TCs and thymomas. Unfortunately, the rate of *KIT* mutations is still only 7 to 9%, despite the high frequency of KIT expression in TC. Four mutations have been described to date: the V560 deletion and L576P substitution found in exon 11, the D820E mutation in exon 17, and the H697Y mutation found in exon 14 [20,22,78].

Angiogenesis significantly influences TET carcinogenesis. Both thymomas and TC overexpress vascular endothelial growth factor (VEGF)-A and VEGFR-1 and -2, although there is little information on the effectiveness of angiogenesis inhibitors in thymic malignancies [79,80]. Low response rates have been observed with bevacizumab [21]. The activity of multikinase inhibitors, particularly sorafenib, and sunitinib, has been emphasized in case reports involving TC [21]. While multikinase inhibitors may have some impact on TC, angiogenesis inhibitors by themselves do not seem to have an effect on either thymomas or TC.

The overexpression of insulin-like growth factor-1 (IGF-1)/IGF-1 receptor (IGF-1R) is a poor prognostic factor in TET. Expression of IGF-1R varies between thymomas (4%) and TC (37%), implying different tumor biologies that might be the subject of targeted therapies [81]. In a retrospective analysis, IGF-1R expression was decreased in types A, AB, and B1 thymomas in comparison with types B2, B3, and TC [82]. A phase II study of cixutumumab, an IGF-1R monoclonal antibody, in 49 patients with previously treated advanced thymic tumors showed limited activity in thymoma (ORR 14%, 95% CI 5–29%) and no effectiveness in TC (ORR 0%, 95% CI 0–26%) [23].

Histone deacetylase (HDAC) inhibitors, in particular the pan-HDAC inhibitor belinostat, were also assessed in TET. A patient with thymoma who participated in a phase I study with belinostat experienced a mild response that lasted for 17 months [83]. A phase II trial with intravenous infusion of belinostat showed only two partial responses in thymomas (ORR 8%, 95% CI 2.3–25.9%) and no responses in TC [25].

The present systematic review has pointed out that the most active agent is lenvatinib, as demonstrated in the REMORA phase II trial. Lenvatinib is a multi-targeted inhibitor of VEGFR, FGFR, RET, c-kit, and other kinases. Further, we can suggest sunitinib as an acceptable second-line therapy for TC [44]. Imatinib demonstrated limited activity in chemotherapy-pretreated patients with TC harboring *KIT* mutations [27,84,85,86]. Everolimus is a potential treatment option for pre-treated patients with TETs when considering durable disease control in a significant proportion of patients with thymomas or TC [49]. Further investigations are underway, including antiangiogenic combinations, for example, ramucirumab with carboplatin and paclitaxel in a first-line setting [87].

## 5. Conclusions

Thymic malignancies are a heterogeneous group of cancers. Heterogeneity and rarity hinder the elaboration of large-scale randomized trials. This systematic review focusing on ICI and targeted therapies shows that ICI and some targeted therapies could be pertinent options for patients with TET not amenable to curative radical treatment when first-line chemotherapy fails. However, it is necessary to be able to define the group of patients most likely to benefit from these molecules by taking into account the benefit/toxicity ratio. Continuous research, not only towards the development of new drugs but also at the microscopic level, should define new targets, and better underline predictors of treatment efficacy and toxicity.

## Figures and Tables

**Table 1:** List of the studies assessing ICI in TET (study design and patient enrollment).

First Author	Year of Publication	Country	Drug	Action	Type of Study	N Patients	M/F	Median Age (Years/Range)
Giaccone [36]	2018	USA	Pembrolizumab	PD-1 inhibitor	Phase II	40	28/12 (70%/30%)	57 (25–80)
Katsuya (PRIMER) [9]	2019	Japan (multi center)	Nivolumab	PD-1 inhibitor	Phase II	15	12/3 (80%/20%)	55 (34–70)
Cho [38]	2019	South Korea (singe center)	Pembrolizumab	PD-1 inhibitor	Phase II	33	33/11 (63.6%/36.4%)	57 (26–78)
Conforti (CAVEATT) [40]	2022	Italy (multi center)	Avelumab+axitinib	PD-1 inhibitor+anti-angiogenic agent	Phase II	32	19/13 (59%/41%)	62 (49–71)
Wang [39]	2022	China	Nivolumab, pembrolizumab, sintilimab, camrelizumab, tislelizumab, toripalimab	PD-1 inhibitors	Retrospective multicentric study	77	48/29 (62.3%/37.7%)	55 (19–84)
Girard (NIVOTHYM) [41]	2023	International multi center	Nivolumab	PD-1 inhibitor	Phase II	55 (49 eligible)	35/20 (64%/36%)	58 (32–82)

ICI: immune checkpoint inhibitors, M: male, F: female.

**Table 2:** List of the studies assessing ICI in TET (objectives and outcomes).

First Author	Median FU Duration (Months)	Primary Endpoint	Histology	Stage	Previous Treatment	ORR	Median PFS (Months)	Median OS (Months)	Grade 3–4 Treatment-Related AE
Giaccone [36]	20	ORR	TC	III (3%), IVa (15%), IVb (82%)	Surgery, radiotherapy, chemotherapy	22.5% (95% CI 10.8–38.5)	4.2 (95% CI 2.9–10.3)	24.9 (15.5–not reached)	Increased AST and ALT (5/13%] patients each, 6 (15%) patients developed severe autoimmune toxicity, including two (5%) patients with myocarditis
Katsuya (PRIMER) [9]	14.1	ORR	TC	Stage III (1/15) IV (14/15)	Surgery, radiotherapy, chemotherapy	0 (95% CI: 0–21.8)	3.8 (95% CI 1.9–7.0)	14.1 (95% CI 11.1-not estimable)	1/15 (AST increase)
Cho [38]	14.9	ORR	7 Thy 26 TC	IVa 48.5%, IVb 51.5%	Surgery, radiotherapy, chemotherapy	21.2% (overall) (10.7 to 37.8)	6.1 (5.3 to 6.9)	Not reported	5 (71.4%) Thymoma and 4 (15.4%) TC: including hepatitis (4; 12.1%), myocarditis (3; 9.1%), myasthenia gravis (2; 6.1%), thyroiditis (1; 3.0%), antineutrophil cytoplasmic antibody–associated glomerulonephritis (1; 3.0%), colitis (1; 3.0%), subacute myoclonus (1; 3.0%)
Conforti (CAVEATT) [40]	22.4	ORR	B3 Thy (3), TC (27), Mixed B3/TC (2)	IVa 3 (9%), IVb 29 (91%)	At least one line of platinum-based chemotherapy	34% (90% CI 21–50)	7.5 (90% CI 3.7–10.0)	26.6 (90% CI 17.0–30.0)	6/32 (19%)
Wang [39]	-	-	TC	III (1.3%), IVa (53.2%), IVb (45.5%)	Surgery, radiotherapy, chemotherapy	36.4%	8.6 (95% CI 4.024–13.109)	35.4 (95% CI 27.628–43.239)	12/77 (15.6%), Grade III most common 4 (5.2%) elevated liver function tests, Grade IV 1 (1.3%) skin rash
Girard (NIVOTHYM) [41]	13.3	PFS rate at 6 months (PFSR-6)	B3 Thy (10), TC (43), other (2)	No M-K stage reported (Not amenable to curative-intent radical treatment)	Prior radical treatment	12% (95% CI 5% to 25%)	6.0 (95% CI 3.1–10.4)	21.3 (95% CI 11.6-not estimable)	G 3/4 in 31 (57%), AEs of grade 4 included 1 neutropenia, 1 immune-mediated transaminitis, 2myocarditis

FU: Follow-up, ORR: objective response rate, Thy: thymoma(s), TC: thymic carcinoma, TET: thymic epithelial tumors, ICI: immune checkpoint inhibitors, PFS: progression-free survival, OS: overall survival, AE: adverse events.

**Table 3:** List of the studies assessing targeted therapies in TET (study design and patient enrollment).

First Author	Year of Publication	Country	Drug	Action	Type of Study	Number of Patients (M/F)	Age
Perrino (Resound trial) [42]	2022	Italy (2 centers)	Regorafenib	VEGFR-PDGFR-FGFR inhibitor	Phase II	19 (8/11)	NR
Guan [47]	2023	China (single center)	Apatinib/anlotinib	VEGFRs, KIT, PDGFRs TKI	Retrospective study	17 (10/7)	59 (35–73)
Remon [43]	2016	France (multicentre)	Sunitinib	VEGFRs, KIT, PDGFRs TKI	Prospective cohort	28 (19/9)	50
Thomas [44]	2015	USA (2 centres)	Sunitinib	VEGFRs, KIT, PDGFRs TKI	Phase II	41 (23/18)	57.5 (31–81)
Antonarelli [45]	2022	Italy (multicentre)	Sunitinib	VEGFRs, KIT, PDGFRs TKI	Retrospective study	20 (10/10)	59 (51–63)
Abu Zaid [46]	2022	USA (single center)	Buparlisib	Pan-PI3K inhibitor	Phase II	14 (4/10)	58 (23–74)
Gubens [48]	2015	USA (2 centres)	Saracatinib	Src inhibitor	Phase II	21 (11/10)	54 (18–84)
Rajan [23]	2014	USA (multicentre)	Cixutumumab	IGF-1R inhibitor	Phase II	49 (26/23)	52 (26–86)
Zucali [49]	2018	Italy (multicentre)	Everolimus	mTOR inhibitor	Phase II	51 (29/22)	55 (36–80)
Giaccone [25]	2011	USA (2 centres)	Belinostat	Pan-HDAC inhibitor	Phase II	41 (20/21)	53 (23–83)
Kurup [50]	2018	USA (2 centres)	Gefitinib	EGFR inhibitor	Phase II	26 (11/15)	NR
Bedano [54]	2008	USA (single center)	Erlotinib/bevacizumab	EGFR inhibitor/VEGFR inhibitor	Phase II	18 (8/10)	NR
Palmieri [51]	2011	Italy (single center)	Imatinib	BCR-ABL TKI	Phase II	15 (10/5)	51 (42–54)
Besse [52]	2018	France (multicentre)	Milciclib	Pan-cyclin d-dependent kinase inhibitor	Phase II (CDKO-125A-006)	72 (NR)	NR
Besse [52]	2018	France (multicentre)	Milciclib	Pan-cyclin d-dependent kinase inhibitor	Phase II (CDKO-125A-007)	30 (NR)	NR
Sato (REMORA) [53]	2020	Japan (multicentre)	Lenvatinib	Multi-targeted inhibitor of VEGFR, FGFR, RET, c-Kit, and other kinases	Phase II	42 (29/13)	55.5 (49–65)

NR: not reported.

**Table 4:** List of the studies assessing targeted therapies in TET (objectives and outcomes).

First Author	Drug	Median FU Duration (Months)	Primary Endpoint	Histology	Stage	Previous Treatment	Median PFS (Months)	Median OS (Months)	ORR	Grade 3–4 Treatment-Related AE
Perrino (Resound trial) [42]	Regorafenib	39.1	8 weeks PFS rate	6 B2/5 B3/8 TC	NR	Platinum-containing chemotherapy	9.6 (95% CI, 3.6–12.8% months)	33.8 (95% CI, 10.2%-not reached)	n/a	52.6% (Hypertension 10.5%, increase in lipase value 5.3%)
Guan [47]	Apatinib/anlotinib	46	n/a	TC	Stage IV	Surgery, chemotherapy	Total 7.9 (6.5–9.3), Apatinib 7 (5.0–9.0), anlotinib 8 (2.7–3.3)	Total 47.0 (35.4–58.6), apatinib 47 (43.7–50.2)	Total 23%, apatinib 30.8%, anlotinib 0%)	Hypertension (3, 23.1%), proteinuria, hand-foot syndrome ( both 2, 15.4%)
Remon [43]	Sunitinib	n/a	n/a	20 TC, 8 T	Stage III and IV	Up to four lines of systemic treatments	Whole population 3.7 (5.4 T, 3.3 TC)	Whole population 15.4 (not reached T, 12.3 TC)	Total 22.2%, Thymomas 28.6%, TC 20%	28.6% (Stomatitis, asthenia, diarrhoea, decline in LVEF)
Thomas [44]	Sunitinib	17	Investigator-assessed best tumour response	25 TC, 16 thymoma	NR	At least one prior platinum-containing chemotherapy	TC: 7.2 (3·4–15·2), thymoma: 8·5 (2.8–11.3)	TC: not reached, thymoma: 15.5 (12.6-undefined)	n/a	Lymphocytopenia (8, 20%), fatigue (8, 20%), oral mucositis (8, 20%). 5 (13%) decreases in LVEF
Antonarelli [45]	Sunitinib	n/a	Median PFS, ORR, median DOR, major treatment-related AEs	12 thymic carcinoma, 6 B3, and 2 B2 thymoma	Stage IV	Platinum refractory	Overall 7.3 (4.5–10.3): 7.3 (4.4-NA) thymoma and 6.8 (2.8–10.3) TC	Not reported	31.6% (12.5%-56.5%)	30% (Asthenia/fatigue 10%)
Abu Zaid [46]	Buparlisib	16.6	ORR	B2 21%/B3 71%	Stage IV	Surgery, radiotherapy, chemotherapy	11.1 (2.9–18.8)	22.5 (10.7–31.3)	7%	Dyspnea (21%), rash (14%), elevated transaminases (14%), cough (7%), pneumonitis (7%), anxiety (7%), fatigue (7%) and hyperglycemia (7%)
Gubens [48]	Saracatinib	Not reported	ORR	12 thymoma, 9 TC		At least one prior chemotherapy	All: 2.5 (1.7–5.7), thymoma 5.3 (1.7–7.8), TC 0.9 (0.9–4.0)	All: 23.1 (7.3–37.5), thymoma 37.5 (12.3-not estimable), TC 6.7 (2.5, 15.0)	0%	Hypophosphatemia 3 (14%), pleural effusion 1 (5%), anemia 1 (5%), hyponatremia 1 (5%), hypoalbuminemia 1 (5%), neutropenia 1 (5%)
Rajan [23]	Cixutumumab	24	ORR	37 thymomas, 12 TC	NR	At least one prior platinum-containing chemotherapy	TC: TTP 1.7 (0.9–2.7)/thymoma: TTP 9.9 (7.3–12.8)	TC: OS 8.4 (4.7–12.8)/thymoma: OS 27.5 (15.0- undefined)	Total 10% (3–22%), thymomas 14% (5–29%, TC 0% (0–26%)	Hyperglycemia (5/10%), lipase elevation (3/6%), weight loss, tumor pain, and hyperuricemia (2 each/4%)
Zucali [49]	Everolimus	25.7	DCR	Thymoma 32, TC 19	Stage III and IV	Systemic therapies	10.1 (6.0–14.2): thymoma 16.6 (9.8–29.8), TC 5.6 (2.6–8.5)	25.7 (16.1-not evaluable): thymoma not reached, TC 14.7 (3.5–24.0)	11.70%	Fourteen patients (28%). Liver toxicity (8%), neutropenia (4%), and metabolic disorders (4%)
Giaccone [25]	Belinostat	Not reported	ORR	25 Thymoma, 16 TC	Stage IV	Surgery, radiotherapy, chemotherapy	Median time to progression 5.8	Thymomas not reached, TC 12.4	Thymomas 8% (2.3–25.9), TC 0% (0–19.4%)	32.5%, QTc prolongation 12.5%
Kurup [50]	Gefitinib	Not reported	ORR	19 Thymomas 7 TC	Stage IV	Systemic therapies	TTP 4 months	NR	3.80%	23%, dyspnea 11.5%, fatigue 3.8%, Anemia/thrombocytopenia 3.8%, myocardial infarction 3.8%
Bedano [54]	Erlotinib/bevacizumab	Not reported	NR	11 Thymoma, 7 TC	NR	NR	NR	Not reached	0%	38.8%, rash 11.1%, dyspnea 11.1%, fatigue 5.5%, pericardial tamponade 5.5%, aortic insufficiency 5.5%
Palmieri [51]	Imatinib	Not reported	NR	12 Thymoma, 3 TC	NR	At least one prior chemotherapy	3 (2–4)	Not reached	0%	None
Besse [52]	Milciclib	Not reported	PFS-3	B3 Thymoma 27.8%, TC 72.2%	NR	One prior chemotherapy	6.83	24.18	3.70%	Neutropenia (8.4%), creatinine, amylase, lipase increase (5.6%), nausea and asthenia (8.3%)
Besse [52]	Milciclib	Not reported	PFS-3	B3 Thymoma 56.7%, TC 43.3%	NR	Multiple chemotherapies	9.76	Not reached	4.20%	Neutropenia (8.4%), creatinine, amylase, lipase increase (5.6%), nausea and asthenia (8.3%)
Sato (REMORA) [53]	Lenvatinib	15.5	ORR	TC	I-IVb (majority stage IVa and IVb)	At least one platinum-based chemotherapy	9.3 (7.7–13.9)	Not reached (16.1-not reached)	38% (25.6–52%)	Hypertension 64%, palmar-plantar erythrodysaesthesia syndrome (7%)

FU: Follow-up, ORR: objective response rate, Thy: thymoma(s), TC: thymic carcinoma, TET: thymic epithelial tumors, PFS: progression-free survival, OS: overall survival, AE: adverse events, NR: not reported.

**Table 5:** Studies investigating the elements of the TME that could be predictive factors of ICI efficacy.

First Author (Year of Publication)	Patients/Tumor Specimens	Histology	TME
Shim (2011) [57]	32	TC	High intensities of stromal CD4+ cells and stromal CD20+ lymphocytes are significantly associated with improved survival in TC
Kim (2019) [61]	31	6 Thymomas–25 TC	A higher fold-change in the percentage of Ki-67+ cells among PD-1+CD8+ T cells 7 days after the first dose (Ki-67_D7/D0_) significantly predicted DCB and prolonged PFS
Blessin (2020) [59]	27	Thymoma (among other types of cancers)	The quantity of TILs influences the likelihood of response to immune checkpoint inhibitors
Kim (2020) [62]	31	6 Thymomas–25 TC	Patients with severe irAEs presented a significantly lower fold increase in the frequency of effector regulatory T (eTreg) cells after anti-PD-1 treatment, a higher ratio of T helper-17 (Th17) and T helper-1 cells at baseline, and a higher percentage of Ki-67+ cells among PD-1+ CD8+ T cells posttreatment
Sato (2020) [58]	42	TC	High mean numbers of stromal CD8+, CD20+, and FOXP3+ cells were significantly associated with favorable prognosis, while high CD204+ cell density tended to be correlated with poor prognosis
Liu (2020) [64]	n/a	Thymoma–TC (among other types of cancers)	A positive relationship between YIF1B expression and immune cell infiltration; YIF1B expression positively correlated with TMB, microsatellite instability, and methylation in some cancer types
Chen (2020) [63]	Not reported	TC (among other types of cancers)	*PRKDC* mutation is one of the significant factors linked to increased TMB, inflamed TME, and greater responsiveness to ICI
Bocchialini (2022) [60]	39	TC	Higher total density of CD3+ and CD8+ TILs in early stages, lower density of CD3+ and CD8+ TILs in advanced TC stages compared to early stages, high densities of stromal CD4+ TILs and CD8+ TILs were associated with improved freedom from recurrence (FFR) and cause-specific survival (CSS), high density of FoxP3+ TILs was associated with improved FFR
Su (2022) [56]	20	Not specified	Higher expression levels of AC138207.2, AC148477.2, AL450270.1 and SNHG8 as well as lower expression levels of AC004466.3, and HOXB-AS1 in TETs samples compared with normal controls; more immunotherapy responders in the low-risk IRL subgroup
Hou (2022) [55]	21	15 Thymomas–6 TC	Higher immune score, higher immune cell infiltration level, and T cell diversity in thymoma; higher stromal score, significantly lower expression of *HMGB1* (a pro-inflammatory cytokine-related gene), which is associated with a dismal prognosis, and higher mutation burden in TC

DCB: Durable clinical benefit, *HMGB1*: high mobility group box 1, ICI: immune checkpoint inhibitors, IRL: immune-related long noncoding RNAs, irAEs: immune-related adverse events, PFS: progression-free survival, *PRKDC*: protein kinase, DNA-activated, catalytic subunit, TME: tumor microenvironment, TC: thymic carcinoma, TILs: tumor-infiltrating lymphocytes, TMB: tumor mutational burden, YIF1B: Yip1 interacting factor homolog B.

**Table 6:** List of ongoing trials assessing immune checkpoint inhibitors in thymic epithelial tumors.

NCT Number	Status	Histology	Intervention	Phase	Enrollment
NCT03463460	Recruiting	Thymic carcinoma	Pembrolizumab/sunitinib malate	II	40
NCT03694002	Active, not recruiting	Thymic carcinoma	Ramucirumab/carboplatine/paclitaxel	II	66
NCT04417660	Recruiting	Thymoma	M7824	II	38
NCT01621568	Active, not recruiting	Thymoma	Sunitinib	II	56
NCT05104736	Recruiting	Thymoma/thymic carcinoma	PT-112	II	53
NCT01306045	Active, not recruiting	Thymic carcinoma	AZD6244/MK-2206/lapatinib/erlotinib/sunitinib	II	647
NCT04667793	Recruiting	Thymoma/thymic carcinoma	Toripalimab and chemotherapy	II	15
NCT03663764	Active, not recruiting	Thymoma/thymic carcinoma	Thymosin a1	II	57
NCT05683886	Recruiting	Thymoma/thymic carcinoma	KC1036	II	30
NCT01025089	Active, not recruiting	Thymoma/thymic carcinoma	Cetuximab, cisplatin, doxorubicin and cyclophosphamide	II	18
NCT03076554	Recruiting	Thymoma/thymic carcinoma	Avelumab	II	55
NCT05832827	Not yet recruiting	Thymic carcinoma	MK-3475/lenvatinib/carboplatin/paclitaxel	II	35
NCT03583086	Active, not recruiting	Thymic carcinoma	VEGFR/ PDGFR dual kinase inhibitor X-82—nivolumab	I/II	88
NCT03134118	Active, not recruiting	Thymoma type B3/thymic carcinoma	Nivolumab	II	55
NCT04925947	Recruiting	Thymic carcinoma	KN046	II	29
NCT04321330	Active, not recruiting	Thymic carcinoma	Atezolizumab	II	34
NCT04710628	Recruiting	Metastatic thymic carcinoma/thymoma Type B3	Pembrolizumab/lenvatinib	II	43

## Data Availability

The following data are publicly available and can be retrieved upon demand: template data collection forms; data extracted from included studies; review protocol. The review is registered in INPLASY (INPLASY202380080).

## Appendix A. The Literature Search Strategy

Subject: immunotherapy and targeted therapies for thymic epithelial tumors
**Medline via l’interface OvidSP** (Epub Ahead of Print, In-Process & Other Non-Indexed Citations, Ovid Medline® Daily and Ovid Medline® 1946-present)
P = exp Thymus Neoplasms/ OR thymus neoplasm*.ti,ab,kw OR thymus cancer*.ti,ab,kw OR Thymus Carcinoma*.ti,ab,kw OR thymus tumour*.ti,ab,kw OR thymus tumor*.ti,ab,kw OR Thymoma*.ti,ab,kw OR thymic neoplasm*.ti,ab,kw OR thymic cancer*.ti,ab,kw OR thymic tumour*.ti,ab,kw OR thymic tumor*.ti,ab,kw OR Thymic Carcinoma*.ti,ab,kw OR Thymic Epithelial Tumor*.ti,ab,kw OR Thymic Epithelial Tumour*.ti,ab,kw
I = exp Angiogenesis Modulating Agents/ OR exp Immunotherapy/ OR immunotherap*.ti,ab,kw OR immunization*.ti,ab,kw OR immunosuppression*.ti,ab,kw OR radioimmunotherap*.ti,ab,kw OR vaccin*.ti,ab,kw OR Cixutumumab.ti,ab,kw,nm OR anti-IGF-1R antibody A12.ti,ab,kw,nm OR Tivantinib.ti,ab,kw,nm OR ARQ 197.ti,ab,kw,nm OR Vorinostat/ OR Vorinostat.ti,ab,kw,nm OR Zolinza.ti,ab,kw,nm OR Ramucirumab.ti,ab,kw,nm OR Ipilimumab/ OR Ipilimumab.ti,ab,kw,nm OR Yervoy.ti,ab,kw,nm OR Anti-CTLA-4.ti,ab,kw,nm OR Tremelimumab.ti,ab,kw,nm OR ticilimumab.ti,ab,kw,nm OR Nivolumab/ OR Nivolumab.ti,ab,kw,nm OR Opdivo.ti,ab,kw,nm OR Pembrolizumab.ti,ab,kw,nm OR lambrolizumab.ti,ab,kw,nm OR Keytruda.ti,ab,kw,nm OR Atezolizumab.ti,ab,kw,nm OR MPDL3280A.ti,ab,kw,nm OR Durvalumab.ti,ab,kw,nm OR Avelumab.ti,ab,kw,nm OR Amatuximab.ti,ab,kw,nm OR MORAb-009.ti,ab,kw,nm OR SS1P.ti,ab,kw,nm OR anetumab ravtansine.ti,ab,kw,nm OR BAY 94-9343.ti,ab,kw,nm OR BNC105P.ti,ab,kw,nm OR BNC-105P.ti,ab,kw,nm OR ADI-PEG20.ti,ab,kw,nm OR pegylated arginine deiminase.ti,ab,kw,nm OR interleukin*.ti,ab,kw,nm OR interferon*.ti,ab,kw,nm OR EZH.ti,ab,kw,nm OR enhancer of zeste homolog.ti,ab,kw,nm OR Immune Checkpoint Inhibitors/ OR Immune Checkpoint Inhibitor*.ti,ab,kw,nm OR Immune Checkpoint Blockade.ti,ab,kw,nm OR PD L1.ti,ab,kw,nm OR PD 1 Inhibitor*.ti,ab,kw,nm OR Tumor Microenvironment/ OR Tumor Microenvironment*.ti,ab,kw OR Tumour Microenvironment*.ti,ab,kw OR Cancer Microenvironment*.ti,ab,kw OR Epidermal Growth Factor/ OR Epidermal Growth Factor.ti,ab,kw OR "HER2/Neu".ti,ab,kw,nm OR "anti-HER-2/neu".ti,ab,kw,nm OR exp Vascular Endothelial Growth Factors/ OR Vascular Endothelial Growth Factor*.ti,ab,kw,nm OR VEGFs.ti,ab,kw,nm OR exp Fibroblast Growth Factors/ OR Fibroblast Growth Factor*.ti,ab,kw,nm OR bFGF.ti,ab,kw,nm OR Tyrosine Protein Kinase Inhibitors/ OR TKI.ti,ab,kw,nm OR Tyrosine kinase inhibitor*.ti,ab,kw,nm OR CTLA-4 Antigen/ OR CTLA 4.ti,ab,kw,nm OR druggable molecular anomal*.ti,ab,kw OR immune-mediated adverse effect*.ti,ab,kw OR genetic marker*.ti,ab,kw OR immune microenvironment.ti,ab,kw OR PI3K.ti,ab,kw,nm
= 1724 (6/02/2023) – 1715 (after duplicate removal)
**SciVerse Scopus**
P = TITLE-ABS-KEY(“thymus neoplasm*” OR “thymus cancer*” OR “Thymus Carcinoma*” OR “thymus tumour*” OR “thymus tumor*” OR Thymoma* OR “thymic neoplasm*” OR "thymic cancer*” OR “thymic tumour*” OR “thymic tumor*” OR “Thymic Carcinoma*” OR “Thymic Epithelial Tumor*” OR “Thymic Epithelial Tumour*”)
I = TITLE-ABS-KEY(immunotherap* OR immunization* OR immunosuppression* OR radioimmunotherap* OR vaccin* OR Cixutumumab OR “anti-IGF-1R antibody A12” OR Tivantinib OR “ARQ 197” OR Vorinostat OR Zolinza OR Ramucirumab OR Ipilimumab OR Yervoy OR “Anti-CTLA-4” OR Tremelimumab OR ticilimumab OR Nivolumab OR Opdivo OR Pembrolizumab OR lambrolizumab OR Keytruda OR Atezolizumab OR “MPDL3280A” OR Durvalumab OR Avelumab OR Amatuximab OR MORAb-009 OR SS1P OR “anetumab ravtansine” OR “BAY 94-9343” OR BNC105P OR BNC-105P OR ADI-PEG20 OR “pegylated arginine deiminase” OR interleukin* OR interferon* OR EZH OR “enhancer of zeste homolog” OR “Immune Checkpoint Inhibitor*” OR “Immune Checkpoint Blockade” OR “PD L1” OR “PD 1 Inhibitor*” OR “Tumor Microenvironment*” OR “Tumour Microenvironment*” OR “Cancer Microenvironment*” OR “Epidermal Growth Factor” OR “HER2/Neu” OR “anti-HER-2/neu” OR “Vascular Endothelial Growth Factor*” OR VEGFs OR “Fibroblast Growth Factor*” OR bFGF OR TKI OR “Tyrosine kinase inhibitor*” OR “CTLA 4” OR “druggable molecular anomal*” OR “immune-mediated adverse effect*” OR “genetic marker*” OR “immune microenvironment” OR PI3K)
= 2747 (6/2/2023)
= 2944 (6/2/2023) merged

## References

[1] Wright C.D. (2008). Management of thymomas. Crit Rev. Oncol. Hematol..

[2] Marx A., Chan J.K., Coindre J.-M., Detterbeck F., Girard N., Harris N.L., Jaffe E.S., Kurrer M.O., Marom E.M., Moreira A.L. (2015). The 2015 World Health Organization classification of tumors of the thymus: Continuity and changes. J. Thorac. Oncol..

[3] Ko R., Shukuya T., Okuma Y., Tateishi K., Imai H., Iwasawa S., Miyauchi E., Fujiwara A., Sugiyama T., Azuma K. (2018). Prognostic factors and efficacy of first-line chemotherapy in patients with advanced thymic carcinoma: A retrospective analysis of 286 patients from NEJ023 study. Oncologist.

[4] Berghmans T., Durieux V., Holbrechts S., Jungels C., Lafitte J.-J., Meert A.-P., Moretti L., Ocak S., Roelandts M., Girard N. (2018). Systemic treatments for thymoma and thymic carcinoma: A systematic review. Lung Cancer.

[5] Girard N., Ruffini E., Marx A., Faivre-Finn C., Peters S., ESMO Guidelines Committee (2015). Thymic epithelial tumours: ESMO Clinical Practice Guidelines for diagnosis, treatment and follow-up. Ann. Oncol..

[6] Ak N., Aydiner A. (2021). Nivolumab treatment for metastatic thymic epithelial tumors. J. Oncol. Pharm. Pract..

[7] Zander T., Aebi S., Rast A.C., Zander A., Winterhalder R., Brand C., Diebold J., Gautschi O. (2016). Response to Pembrolizumab in a patient with relapsing thymoma. J. Thorac. Oncol..

[8] Uchida N., Fujita K., Okamura M., Nakatani K., Mio T. (2019). The clinical benefits of immune checkpoint inhibitor for thymic carcinomas approximately experience of single public hospital in Japan approximately. Respir. Med. Case Rep..

[9] Katsuya Y., Horinouchi H., Seto T., Umemura S., Hosomi Y., Satouchi M., Nishio M., Kozuki T., Hida T., Sukigara T. (2019). Single-arm, multicentre, phase II trial of nivolumab for unresectable or recurrent thymic carcinoma: PRIMER study. Eur. J. Cancer.

[10] Yang Y., Ding L., Wang P. (2016). Dramatic response to anti-PD-1 therapy in a patient of squamous cell carcinoma of thymus with multiple lung metastases. J. Thorac. Dis..

[11] Yang P.-C., Guo J.-C., Hsieh M.-S., Lin C.-C., Hsu C.-H. (2018). Response to nivolumab as salvage therapy in a patient with thymic carcinoma. J. Thorac. Oncol..

[12] Girard N. (2019). Immune checkpoints in thymic epithelial tumors: Challenges and opportunities. Immuno-Oncol. Technol..

[13] Nivolumab in Patients with Type B3 Thymoma and Thymic Carcinoma (NIVOTHYM). https://clinicaltrials.gov/ct2/show/NCT03134118?term=NCT03134118&draw=2&rank=1.

[14] Palmieri G., Marino M., Salvatore M., Budillon A., Meo G., Caraglia M., Montella L. (2007). Cetuximab is an active treatment of metastatic and chemorefractory thymoma. Front. Biosci..

[15] Farina G., Garassino M.C., Gambacorta M., La Verde N., Gherardi G., Scanni A. (2007). Response of thymoma to cetuximab. Lancet Oncol..

[16] Christodoulou C., Murray S., Dahabreh J., Petraki K., Nikolakopoulou A., Mavri A., Skarlos D. (2008). Response of malignant thymoma to erlotinib. Ann. Oncol..

[17] Pedersini R., Vattemi E., Lusso M.R., Mazzoleni G., Ebner H., Graiff C. (2008). Erlotinib in advanced well-differentiated thymic carcinoma with overexpression of EGFR: A case report. Tumori J..

[18] Nakagiri T., Funaki S., Kadota Y., Takeuchi Y., Shiono H., Akashi A., Okumura M. (2014). Does gefitinib have effects on EGFR mutation-positive thymoma? Case report of thymoma recurrence. Ann. Thorac. Cardiovasc. Surg..

[19] Giaccone G., Rajan A., Ruijter R., Smit E., van Groeningen C., Hogendoorn P.C. (2009). Imatinib mesylate in patients with WHO B3 thymomas and thymic carcinomas. J. Thorac. Oncol..

[20] Ströbel P., Hartmann M., Jakob A., Mikesch K., Brink I., Dirnhofer S., Marx A. (2004). Thymic carcinoma with overexpression of mutated KIT and the response to imatinib. N. Engl. J. Med..

[21] Buti S., Donini M., Sergio P., Garagnani L., Schirosi L., Passalacqua R., Rossi G. (2011). Impressive response with imatinib in a heavily pretreated patient with metastatic c-KIT mutated thymic carcinoma. J. Clin. Oncol..

[22] Bisagni G., Rossi G., Cavazza A., Sartori G., Gardini G., Boni C. (2009). Long lasting response to the multikinase inhibitor bay 43–9006 (Sorafenib) in a heavily pretreated metastatic thymic carcinoma. J. Thorac. Oncol..

[23] Rajan A., Carter C.A., Berman A., Cao L., Kelly R.J., Thomas A., Khozin S., Chavez A.L., Bergagnini I., Scepura B. (2014). Cixutumumab for patients with recurrent or refractory advanced thymic epithelial tumours: A multicentre, open-label, phase 2 trial. Lancet Oncol..

[24] Wheler J.J., Hong D., Swisher S.G., Falchook G.S., Tsimberidou A.M., Helgason T., Naing A., Stephen B., Janku F., Stephens P.J. (2013). Thymoma patients treated in a phase I clinic at MD Anderson Cancer Center: Responses to mTOR inhibitors and molecular analyses. Oncotarget.

[25] Giaccone G., Rajan A., Berman A., Kelly R.J., Szabo E., Lopez-Chavez A., Trepel J., Lee M.-J., Cao L., Espinoza-Delgado I. (2011). Phase II study of belinostat in patients with recurrent or refractory advanced thymic epithelial tumors. J. Clin. Oncol..

[26] Azad A., Herbertson R.A., Pook D., White S., Mitchell P.L., Tebbutt N.C. (2009). Motesanib diphosphate (AMG 706), an oral angiogenesis inhibitor, demonstrates clinical efficacy in advanced thymoma. Acta Oncol..

[27] Ströbel P., Bargou R., Wolff A., Spitzer D., Manegold C., Dimitrakopoulou-Strauss A., Strauss L., Sauer C., Mayer F., Hohenberger P. (2010). Sunitinib in metastatic thymic carcinomas: Laboratory findings and initial clinical experience. Br. J. Cancer.

[28] Chuah C., Lim T.H., Lim A.S.T., Tien S.L., Lim C.H., Soong R., Lee F., Linn Y.C., Goh Y.T., Cheah F.K. (2006). Dasatinib induces a response in malignant thymoma. J. Clin. Oncol..

[29] Neuhaus T., Luyken J. (2012). Long lasting efficacy of sorafenib in a heavily pretreated patient with thymic carcinoma. Target. Oncol..

[30] Hinshaw D.C., Shevde L.A. (2019). The tumor microenvironment innately modulates cancer progression. Cancer Res..

[31] Agrafiotis A.C., Siozopoulou V., Hendriks J.M.H., Pauwels P., Koljenovic S., Van Schil P.E. (2022). Tumor Microenvironment in Thymic Epithelial Tumors: A Narrative Review. Cancers.

[32] Ohm B., Jungraithmayr W. (2023). Balancing the Risk of Adverse Events against the Efficacy of Immunotherapy in Advanced Thymic Epithelial Tumors. Cancers.

[33] Masaoutis C., Palamaris K., Kokkali S., Levidou G., Theocharis S. (2022). Unraveling the Immune Microenvironment of Thymic Epithelial Tumors: Implications for Autoimmunity and Treatment. Int. J. Mol. Sci..

[34] Simon R., Peace K.E. (1987). Design, Analysis and Reporting of Cancer Clinical Trials. Biopharmaceutical Statistics for Drug Development.

[35] Simon R. (1989). Optimal two-stage designs for phase II clinical trials. Control. Clin. Trials.

[36] Giaccone G., Kim C., Thompson J., McGuire C., Kallakury B., Chahine J.J., Manning M., Mogg R., Blumenschein W.M., Tan M.T. (2018). Pembrolizumab in patients with thymic carcinoma: A single-arm, single-centre, phase 2 study. Lancet Oncol..

[37] Giaccone G., Kim C. (2021). Durable Response in Patients with Thymic Carcinoma Treated with Pembrolizumab After Prolonged Follow-Up. J. Thorac. Oncol..

[38] Cho J., Kim H.S., Ku B.M., Choi Y.-L., Cristescu R., Han J., Sun J.-M., Lee S.-H., Ahn J.S., Park K. (2019). Pembrolizumab for patients with refractory or relapsed thymic epithelial tumor: An open-label phase II trial. J. Clin. Oncol..

[39] Wang W., Lin G., Hao Y., Guan Y., Zhang Y., Xu C., Wang Q., Wang D., Jiang Z., Cai J. (2022). Treatment outcomes and prognosis of immune checkpoint inhibitors therapy in patients with advanced thymic carcinoma: A multicentre retrospective study. Eur. J. Cancer.

[40] Conforti F., Zucali P.A., Pala L., Catania C., Bagnardi V., Sala I., Della Vigna P., Perrino M., Zagami P., Corti C. (2022). Avelumab plus axitinib in unresectable or metastatic type B3 thymomas and thymic carcinomas (CAVEATT): A single-arm, multicentre, phase 2 trial. Lancet Oncol..

[41] Girard N., Aix S.P., Cedres S., Berghmans T., Burgers S., Toffart A.-C., Popat S., Janssens A., Gervais R., Hochstenbag M. (2023). Efficacy and safety of nivolumab for patients with pre-treated type B3 thymoma and thymic carcinoma: Results from the EORTC-ETOP NIVOTHYM phase II trial. ESMO Open.

[42] Perrino M., De Pas T., Bozzarelli S., Giordano L., De Vincenzo F., Conforti F., Digiacomo N., Cordua N., D’Antonio F., Borea F. (2022). Resound Trial: A phase 2 study of regorafenib in patients with thymoma (type B2-B3) and thymic carcinoma previously treated with chemotherapy. Cancer.

[43] Remon J., Girard N., Mazieres J., Dansin E., Pichon E., Greillier L., Dubos C., Lindsay C.R., Besse B. (2016). Sunitinib in patients with advanced thymic malignancies: Cohort from the French RYTHMIC network. Lung Cancer.

[44] Thomas A., Rajan A., Berman A., Tomita Y., Brzezniak C., Lee M.-J., Lee S., Ling A., Spittler A.J., Carter C.A. (2015). Sunitinib in patients with chemotherapy-refractory thymoma and thymic carcinoma: An open-label phase 2 trial. Lancet Oncol..

[45] Antonarelli G., Corti C., Zucali P.A., Perrino M., Manglaviti S., Russo G.L., Varano G.M., Salvini P., Curigliano G., Catania C. (2022). Continuous sunitinib schedule in advanced platinum refractory thymic epithelial neoplasms: A retrospective analysis from the ThYmic MalignanciEs (TYME) Italian collaborative group. Eur. J. Cancer.

[46] Abu Zaid M.I., Radovich M., Althouse S., Liu H., Spittler A.J., Solzak J., Badve S., Loehrer P.J. (2022). A phase II study of buparlisib in relapsed or refractory thymomas. Front. Oncol..

[47] Guan Y., Gu X., Si J., Xiang J., Wei J., Hao Y., Wang W., Sun Y. (2023). The efficacy of small molecule anti-angiogenic drugs in previously treated Thymic carcinoma. BMC Cancer.

[48] Gubens M.A., Burns M., Perkins S.M., Pedro-Salcedo M.S., Althouse S.K., Loehrer P.J., Wakelee H.A. (2015). A phase II study of saracatinib (AZD0530), a Src inhibitor, administered orally daily to patients with advanced thymic malignancies. Lung Cancer.

[49] Zucali P.A., De Pas T., Palmieri G., Favaretto A., Chella A., Tiseo M., Caruso M., Simonelli M., Perrino M., De Vincenzo F. (2018). Phase II Study of Everolimus in Patients with Thymoma and Thymic Carcinoma Previously Treated with Cisplatin-Based Chemotherapy. J. Clin. Oncol..

[50] Kurup A., Burns M., Dropcho S., Pao W., Loehrer P.J. (2005). Phase II study of gefitinib treatment in advanced thymic malignancies. J. Clin. Oncol..

[51] Palmieri G., Marino M., Buonerba C., Federico P., Conti S., Milella M., Petillo L., Evoli A., Lalle M., Ceribelli A. (2012). Imatinib mesylate in thymic epithelial malignancies. Cancer Chemother. Pharmacol..

[52] Besse B., Garassino M.C., Rajan A., Novello S., Mazieres J., Weiss G.J., Kocs D.M., Barnett J.M., Davite C., Crivori P. (2018). Efficacy of milciclib (PHA-848125AC), a pan-cyclin d-dependent kinase inhibitor, in two phase II studies with thymic carcinoma (TC) and B3 thymoma (B3T) patients. J. Clin. Oncol..

[53] Sato J., Satouchi M., Itoh S., Okuma Y., Niho S., Mizugaki H., Murakami H., Fujisaka Y., Kozuki T., Nakamura K. (2020). Lenvatinib in patients with advanced or metastatic thymic carcinoma (REMORA): A multicentre, phase 2 trial. Lancet Oncol..

[54] Bedano P.M., Perkins S., Burns M., Kessler K., Nelson R., Schneider B.P., Risley L., Dropcho S., Loehrer P.J. (2008). A phase II trial of erlotinib plus bevacizumab in patients with recurrent thymoma or thymic carcinoma. J. Clin. Oncol..

[55] Hou X., Lin S., Liu Y., Wang K., Yu Z., Jia J., Yu J., Zheng W., Bai J., Chang L. (2022). Analysis of the tumor microenvironment and mutation burden identifies prognostic features in thymic epithelial tumors. Am. J. Cancer Res..

[56] Su Y., Ou Y., Chen Y., Ma X. (2022). Construction of immune-related LncRNAs classifier to predict prognosis and immunotherapy response in thymic epithelial tumors. Biosci. Rep..

[57] Shim H.S., Byun C.S., Bae M.K., Lee C.Y., Park I.K., Kim D.J., Chung K.Y., Lee J.G. (2011). Prognostic effect of stromal lymphocyte infiltration in thymic carcinoma. Lung Cancer.

[58] Sato J., Kitano S., Motoi N., Ino Y., Yamamoto N., Watanabe S., Ohe Y., Hiraoka N. (2020). CD20+ tumor-infiltrating immune cells and CD204+ M2 macrophages are associated with prognosis in thymic carcinoma. Cancer Sci..

[59] Blessin N.C., Spriestersbach P., Li W., Mandelkow T., Dum D., Simon R., Hube-Magg C., Lutz F., Viehweger F., Lennartz M. (2020). Prevalence of CD8+ cytotoxic lymphocytes in human neoplasms. Cell. Oncol..

[60] Bocchialini G., Schiefer A.I., Müllauer L., Thanner J., Bauer J., Thaler F., Laggner M., Veraar C., Klepetko W., Hötzenecker K. (2022). Tumour immune microenvironment in resected thymic carcinomas as a predictor of clinical outcome. Br. J. Cancer.

[61] Kim K.H., Cho J., Ku B.M., Koh J., Sun J.-M., Lee S.-H., Ahn J.S., Cheon J., Min Y.J., Park S.-H. (2019). The First-week Proliferative Response of Peripheral Blood PD-1+CD8+ T Cells Predicts the Response to Anti-PD-1 Therapy in Solid Tumors. Clin. Cancer Res..

[62] Kim K.H., Hur J.Y., Cho J., Ku B.M., Koh J., Koh J.Y., Sun J.-M., Lee S.-H., Ahn J.S., Park K. (2020). Immune-related adverse events are clustered into distinct subtypes by T-cell profiling before and early after anti-PD-1 treatment. OncoImmunology.

[63] Chen Y., Li Y., Guan Y., Huang Y., Lin J., Chen L., Li J., Chen G., Pan L.K., Xia X. (2020). Prevalence of PRKDC mutations and association with response to immune checkpoint inhibitors in solid tumors. Mol. Oncol..

[64] Liu J., Chen Z., Zhao P., Li W. (2020). Prognostic and immune regulating roles of YIF1B in Pan-Cancer: A potential target for both survival and therapy response evaluation. Biosci. Rep..

[65] Thapa P., Farber D.L. (2019). The Role of the Thymus in the Immune Response. Thorac. Surg. Clin..

[66] Arbour K.C., Naidoo J., Steele K.E., Ni A., Moreira A.L., Rekhtman N., Robbins P.B., Karakunnel J., Rimner A., Huang J. (2017). Expression of PD-L1 and other immunotherapeutic targets in thymic epithelial tumors. PLoS ONE.

[67] Tateo V., Manuzzi L., De Giglio A., Parisi C., Lamberti G., Campana D., Pantaleo M. (2020). Immunobiology of Thymic Epithelial Tumors: Implications for Immunotherapy with Immune Checkpoint Inhibitors. Int. J. Mol. Sci..

[68] Herbst R.S., Soria J.C., Kowanetz M., Fine G.D., Hamid O., Gordon M.S., Sosman J.A., McDermott D.F., Powderly J.D., Gettinger S.N. (2014). Predictive correlates of response to the anti–PD-L1 antibody MPDL3280A in cancer patients. Nature.

[69] Mazzaschi G., Madeddu D., Falco A., Bocchialini G., Goldoni M., Sogni F., Armani G., Lagrasta C.A., Lorusso B., Mangiaracina C. (2018). Low PD-1 expression in cytotoxic CD8 + tumor-infiltrating lymphocytes confers an immune-privileged tissue microenvironment in NSCLC with a prognostic and predictive value. Clin. Cancer Res..

[70] Yokoyama S., Miyoshi H., Nakashima K., Shimono J., Hashiguchi T., Mitsuoka M., Takamori S., Akagi Y., Ohshima K. (2016). Prognostic value of programmed death ligand 1 and programmed death 1 expression in thymic carcinoma. Clin. Cancer Res..

[71] Graab P., Bock C., Weiss K., Hirth A., Koller N., Braner M., Jung J., Loehr F., Tampé R., Behrends C. (2019). Lysosomal targeting of the ABC transporter TAPL is determined by membrane-localized charged residues. J. Biol. Chem..

[72] Petrini I., Meltzer P.S., Kim I.-K., Lucchi M., Park K.-S., Fontanini G., Gao J., A Zucali P., Calabrese F., Favaretto A. (2014). A specific missense mutation in GTF2I occurs at high frequency in thymic epithelial tumors. Nat. Genet..

[73] Yoh K., Nishiwaki Y., Ishii G., Goto K., Kubota K., Ohmatsu H., Niho S., Nagai K., Saijo N. (2008). Mutational status of EGFR and KIT in thymoma and thymic carcinoma. Lung Cancer.

[74] Yamaguchi H., Soda H., Kitazaki T., Tsukamoto K., Hayashi T., Kohno S. (2006). Thymic carcinoma with epidermal growth factor receptor gene mutations. Lung Cancer.

[75] Suzuki E., Sasaki H., Kawano O., Endo K., Haneda H., Yukiue H., Kobayashi Y., Yano M., Fujii Y. (2006). Expression and mutation statuses of epidermal growth factor receptor in thymic epithelial tumors. Jpn. J. Clin. Oncol..

[76] Henley J.D., Cummings O.W., Loehrer P.J. (2004). Tyrosine kinase receptor expression in thymomas. J. Cancer Res. Clin. Oncol..

[77] Pan C.C., Chen P.C., Chiang H. (2004). KIT (CD117) is frequently overexpressed in thymic carcinomas but is absent in thymomas. J. Pathol..

[78] Girard N., Shen R., Guo T., Zakowski M.F., Heguy A., Riely G.J., Huang J., Lau C., Lash A.E., Ladanyi M. (2009). Comprehensive genomic analysis reveals clinically relevant molecular distinctions between thymic carcinomas and thymomas. Clin. Cancer Res..

[79] Cimpean A.M., Raica M., Encica S., Cornea R., Bocan V. (2008). Immunohistochemical expression of vascular endothelial growth factor A (VEGF), and its receptors (VEGFR1, 2) in normal and pathologic conditions of the human thymus. Ann. Anat. Anat. Anz..

[80] Sasaki H., Yukiue H., Kobayashi Y., Nakashima Y., Moriyama S., Kaji M., Kiriyama M., Fukai I., Yamakawa Y., Fujii Y. (2001). Elevated serum vascular endothelial growth factor and basic fıbroblast growth factor levels in patients with thymic epithelial neoplasms. Surg. Today.

[81] Girard N., Teruya-Feldstein J., Payabyab E.C., Riely G.J., Rusch V.W., Kris M.G., Zakowski M.F. (2010). Insulin-like growth factor-1 receptor expression in thymic malignancies. J. Thorac. Oncol..

[82] Zucali P.A., Petrini I., Lorenzi E., Merino M., Cao L., Di Tommaso L., Lee H.S., Incarbone M., Walter B.A., Simonelli M. (2010). Insulin-like growth factor-1 receptor and phosphorylated AKT-serine 473 expression in 132 resected thymomas and thymic carcinomas. Cancer.

[83] Steele N.L., Plumb J.A., Vidal L., Tjørnelund J., Knoblauch P., Rasmussen A., Ooi C.E., Buhl-Jensen P., Brown R., Evans T.R.J. (2008). A phase 1 pharmacokinetic and pharmacodynamic study of the histone deacetylase inhibitor belinostat in patients with advanced solid tumors. Clin. Cancer Res..

[84] Petrini I., Zucali P.A., Lee H.S., Pineda M.A., Meltzer P.S., Walter-Rodriguez B., Roncalli M., Santoro A., Wang Y., Giaccone G. (2010). Expression and mutational status of c-kit in thymic epithelial tumors. J. Thorac. Oncol..

[85] Hirai F., Edagawa M., Shimamatsu S., Toyozawa R., Toyokawa G., Nosaki K., Yamaguchi M., Seto T., Twakenoyama M., Ichinose Y. (2016). c-kit mutation-positive advanced thymic carcinoma successfully treated as a mediastinal gastrointestinal stromal tumor: A case report. Mol. Clin. Oncol..

[86] Hagemann I.S., Govindan R., Javidan-Nejad C., Pfeifer J.D., Cottrell C.E. (2014). Stabilization of disease after targeted therapy in a thymic carcinoma with KIT mutation detected by clinical next-generation sequencing. J. Thorac. Oncol..

[87] Imbimbo M., Vitali M., Fabbri A., Ottaviano M., Pasello G., Petrini I., Palmieri G., Berardi R., Zucali P., Ganzinelli M. (2018). RELEVENT Trial: Phase II Trial of Ramucirumab, Carboplatin, and Paclitaxel in Previously Untreated Thymic Carcinoma/B3 Thymoma with Area of Carcinoma. Clin. Lung Cancer.

